# Removal of Malachite Green Dye from Aqueous Solution by a Novel Activated Carbon Prepared from Baobab Seeds Using Chemical Activation Method

**DOI:** 10.3390/molecules30020407

**Published:** 2025-01-19

**Authors:** Samah Daffalla

**Affiliations:** Department of Environment and Agricultural Natural Resources, College of Agricultural and Food Sciences, King Faisal University, P.O. Box 400, Al-Ahsa 31982, Saudi Arabia; sbalal@kfu.edu.sa

**Keywords:** malachite green, baobab seeds, activated carbon, chemical activation, oxidation process, regeneration studies

## Abstract

Two activated carbons were synthesized from baobab seeds (BSs) using two activators, sulfuric acid (BS-AAC) and sodium hydroxide (BS-BAC), for dye removal from aqueous solutions. Malachite green (MG) was used as a model dye. SEM, FTIR, TGA, and surface area were used to characterize the feedstock and synthesis activated carbons. According to the SEM results, the surface morphology differed significantly from that of the raw material due to the many pores created by activating agents during carbonization. Various surface groups existed on the activated carbon surface as shown by FTIR analysis. An oxidation process utilizing hydrogen peroxide (H_2_O_2_) was investigated for MG. Various reaction parameters such as pH value, H_2_O_2_ concentration, and activated carbon dosage were investigated for the oxidative degradation of MG. By using BS-AAC and BS-BAC, 97.9% and 78% dye degradation efficiency in aqueous solutions, respectively, was achieved under optimal conditions. This study reveals that MG dye degradation increases with solution pH, making BS-AAC and BS-BAC ineffective at low pH values. However, degradation declines above pH 6. Based on the BS-AAC data, MG removal kinetics were fitted with a first-order kinetic model, while BS-BAC data were fitted with a second-order kinetic model. It was demonstrated that activating baobab with sulfuric acid can form a novel activated carbon that can quickly remove MG from aqueous solutions. The results showed that the removal of malachite green was over 89% for AC-AAC and 77% for AC-BAC, even after four regeneration cycles.

## 1. Introduction

The high growth of population growth, along with industrial progress, leads to a high bad influence on the environment. The contamination of water is highly affected by the great amounts of industrial waste. Various industries, such as cosmetics, fabric, and plastics, discharge synthetic organic and inorganic pollutants into water streams. The discharging of these pollutants into the environment is a highly noteworthy environmental issue. Moreover, the abundance of dyes in marine systems reduces the dispersion of light and photosynthesis. Additionally, because it was biotransformation products, it can be extremely harmful to aquatic life [1,2,3]. Therefore, it is very important to remove these pollutants from industrial effluents before they are released into water bodies. Among the most common cationic dyes is malachite green (MG), which is broadly used in many industries. This dye is harmful to the environment and biology. Several methods such as adsorption, advanced oxidation processes (AOPs), nanofiltration, electrochemical membrane separations, etc., were used to remove dyes from wastewater [1,2]. AOPs are often regarded as the best approach for treating organic wastewater among several chemical treatments. AOPs have the benefits of high mineralization efficiency, a quick oxidation-reaction rate, and no secondary pollution. Various chemicals are used in these processes, including sodium hypochloride, hydrogen peroxide (H_2_O_2_), ozone and H_2_O_2_, potassium ferrate (VI), potassium permanganate, etc. Hydroxyl radicals can be generated from H_2_O_2_ using either homogeneous (such as the Fenton reaction) or heterogeneous catalysts (such as supported metal catalysts, metal oxides, graphite, and activated carbon (AC)) [4]. One potential technique for degrading contaminants in aqueous media is the use of strong oxidants like hydroxyl radicals (OH•). Numerous articles discuss the oxidation of various dyes from aqueous solutions by Fenton reactions, photocatalytic oxidation, electrochemical oxidation reactions, ozone, ultrasonic, and other AOPs [3,5,6,7,8,9,10].

AC is a carbonaceous substance with an amorphous structure that has a high degree of porosity and a well-developed surface area. Carbonization and activation are basic procedures for the production of AC [11,12]. There are several factors that affect the activation process, including the type of activating agent used (acid, base, or neutral), impregnation ratio, and heating method [11]. The majority of researchers have found AC to be successful in removing many contaminants from wastewater. AC is often created from limited resources such as coal, lignite, peat, and petroleum residual materials, which are costly and need extensive regeneration. The rising demand and needs have resulted in the development of less expensive, more environmentally friendly, and more sustainable materials for the manufacturing of AC from the thermal treatment of biomass [13]. Previous studies show that different ACs derived from biomass, such as waste orange and lemon peels [12], waste coffee grounds [14], and mangosteen peel wastes [15], have been utilized to remove dyes from contaminated aqueous systems. Because of its affordability, abundance, and sustainability, cellulose-based material derived from agriculture waste has long been regarded as a suitable resource for organic synthesis [16]. Although AC adsorption is effective in removing organic compounds, it is prone to saturation during the process, which requires regeneration or replacement of the AC completely. A combination of adsorption and heterogeneous catalysis could be an attractive approach to wastewater treatment.

In Africa, baobab trees are known for their woody, indehiscent fruits that are oval and pendulous and have long stalks. Usually, there are two to eight locules in each fruit, each containing up to eight rows of large brown seeds. The fruit walls are lined with fibrous strands that encase seeds in yellowish-white pulp. A traditional method in Sudan is to chew, swallow, or crush fruit pulp into drinks. Fruit pulp contains tartaric acid and is used to season fruits [17]. This work is unique in that it synthesizes AC from a novel precursor, baobab seeds, that contains a high natural fiber content. In addition to being environmentally friendly and low-cost, baobab seeds are green, non-toxic, and non-carcinogenic. Hence, in this research, the influences of the physical and chemical properties of the synthesis of a novel AC from baobab seeds using base and acid activating agents were investigated. A UV-Vis spectrophotometer was used to study the oxidation of dye from aqueous solutions using high-efficiency baobab-seed ACs and hydrogen peroxide. MG was used as the model dye. The effect of H_2_O_2_ dose, activated carbon dose, and pH was investigated. In addition, the kinetics of oxidation of MG dye were tested. Furthermore, the regeneration and reuse of AC were studied.

## 2. Results and Discussions

### 2.1. Characterization

The BS-AAC showed the highest yield (34.5%), followed by BS-BAC (30.9%).

#### 2.1.1. SEM Characterization

**Figure 1:**
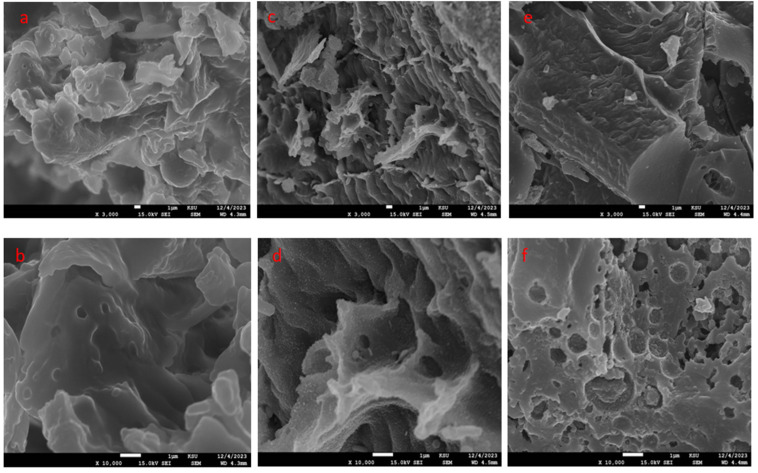
SEM analysis of BS (**a**,**b**), BS-AAC (**c**,**d**), and BS-BAC (**e**,**f**) magnified 3000 and 10,000.

The morphological structure of BSs and the produced BS-ACs employing chemical activation techniques with H_2_SO_4_ and NaOH were both observed using SEM. As can be seen from the SEM images in Figure 1, the surface morphology of the baobab-seed activated carbons (Figure 1c,f) differed significantly from the raw material in (Figure 1a,b) as a result of the many pores created by activating agents during carbonization. The results indicated that chemicals were responsible for influencing the topography of carbon surfaces. The surface of the baobab seed biomass was heterogeneous and appeared to have few visible pores. This may be due to the presence of different functional groups on this surface (Figure 1a,b). Undulating surfaces with a porous structure appeared after the H_2_SO_4_ activation (Figure 1c,d) due to its dehydration effect and the oxidation of organic compounds in the carbonization step [18,19]. In the case of activated carbon, the external surface is cracked and creviced and has some grainy deposits in a variety of sizes in large holes after activation. Vunain et al. [18] observed a similar morphological structure for activated carbon derived from baobab fruit shells using phosphoric acid as an activating agent. Based on Figure 1e,f, different mechanisms of reaction are demonstrated in the morphology of BS-BAC with pores of different sizes. It has been demonstrated that the pores and cavities developed in H_2_SO_4_ (Figure 1c) were highly determinate when compared to NaOH (Figure 1e). Further, the H_2_SO_4_ micrograph has the highest number of pores compared to the NaOH micrograph. EDS analysis was used to investigate the elemental composition present in baobab seeds and their activated carbons. Table 1 shows the presence of different elements corresponding to carbon, oxygen, and silicon on the materials for raw baobab seeds, BS-AAC and BS-BAC. These EDS peaks are attributed to the quartz and Calcite composition of the raw material and its activated carbon [19]. The increase in carbon content and decrease in silica content after pyrolysis of a sample are likely due to the selective removal or volatilization of silica-containing components during high-temperature treatment [20]. Considering these facts, it can be concluded that the activated carbons prepared from baobab seeds exhibit an appropriate morphology for the oxidation of dyes.

#### 2.1.2. FTIR Characterization

**Figure 2:**
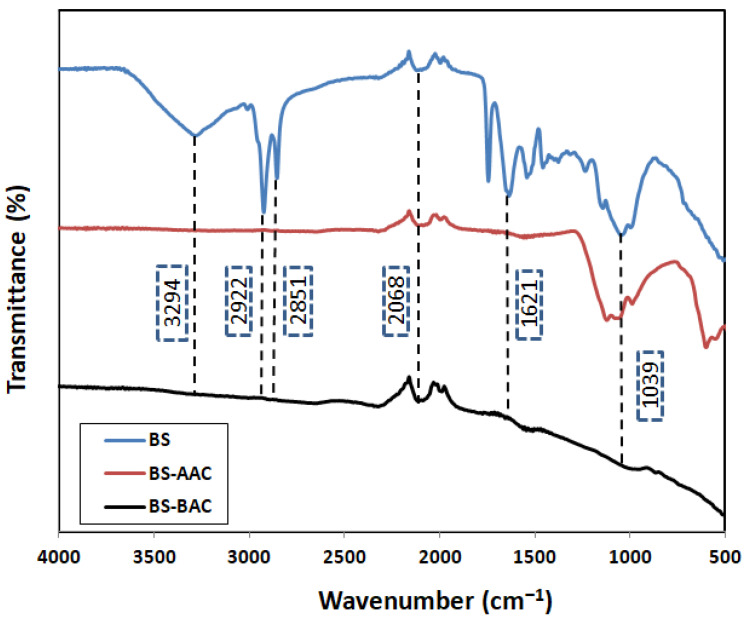
FTIR spectra of BS, BS-AAC, and BS-BAC.

FTIR analysis of the feedstock and developed ACs (BS-AAC and BS-BAC) is shown in Figure 2. Compared to that of BS-AC, the raw BS spectrum had more absorbent bands. For BS, the presence of hydrogen-bound (O–H) groups of cellulose, pectin, and lignin was attributed to the peak that was seen at 3294 cm^−1^ [19]. A symmetric stretching of (C–H) and an alkyl group of (–CH_2_) can be attributed to the peaks at around 2922 and 2851 cm^−1^. A band of 3068 cm^−1^ was observed due to (C≡N) stretching. Ketones, aldehydes, lactones, and carboxyls contribute to the peak at 1621 cm^−1^, which corresponds to olefinic stretching (C=C) vibration [19], which disappeared after the activation process. A comparatively low-intensity band at around 1412 cm^−1^ was created by the presence of (C=C) stretching in the aromatic skeleton and ester molecules. In BS, hydroxyl, carbonyl, and carboxyl groups are the major functional groups. In BS-ACC and BS-BAC, a comparable FTIR spectrum is available, showing different types of surface functional groups. The breakdown of carboxyl groups is the primary cause of the decrease in relative intensity seen for both activated carbons at heights between 4000 cm^−1^ and 2000 cm^−1^. BS-AAC shows a stronger band than BS-BAC, which also reveals the presence of a large number of functional groups in BS-AAC. For BS-ACC, the peaks around 1099 and 1039 cm^−1^ can be attributed to the presence of (C–O) extending groups. As shown in Figure 2, a peak at 987 cm^−1^ was also observed, which was explained to be the peak of the alkene functional group on the BS-AAC surface. Every peak was located and given a label based on comparable accounts found in the literature [19,21].

#### 2.1.3. Surface Area and Pore Size Analysis

**Figure 3:**
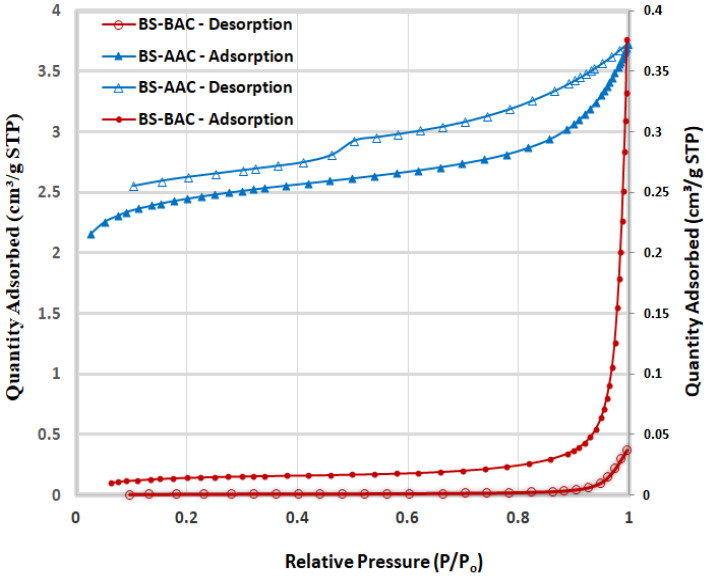
Hysteresis loops of BS-AAC and BS-BAC.

BS, BS-AAC, and BS-BAC hysteresis loops are shown in Figure 3. As classified by IUPAC, BS-BAC exhibited hysteresis loops H3 and H4, which indicates a macroporous and mesoporous structure [22]. By contrast, BS-AAC exhibited type IV isotherms with an H3 hysteresis loop, which is typical of mesoporous and microporous materials. Indeed, the size of the mesopores grows and additional micropores form as the pyrolysis temperature rises above 550 °C [23]. A BET analysis also revealed that BS-BAC had low surface areas (11.27 m^2^/g) and that BS-AAC had the largest surface area (174.4 m^2^/g) (Table 2). The high mesoporous content in BS-AAC suggests that the treatment process is appropriate, as it offers many active sites for the breakdown of contaminants.

**Figure 4:**
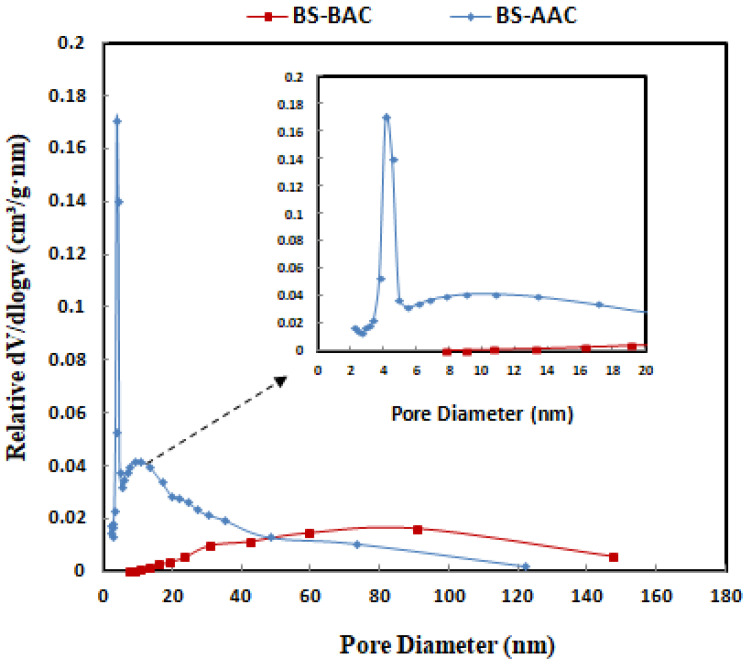
Pore size distribution of BS-AAC and BS-BAC.

The pore size distribution of the BS-AAC and BS-BAC samples is presented in Figure 4. The pore size distributions of these adsorbents were calculated from the N_2_ adsorption isotherm following the Barrett–Joyner–Halenda (BJH) method with calibration. As shown in Figure 4, the distribution of the pore sizes of the BS-AAC and BS-BAC samples can be seen. There was a relatively broad pore distribution in both the BS and BS-BAC samples. On the other hand, the pore distribution for the BS-AAC sample shifted into mesopores with a center at 4 and 2.7 nm.

#### 2.1.4. TGA Analysis

**Figure 5:**
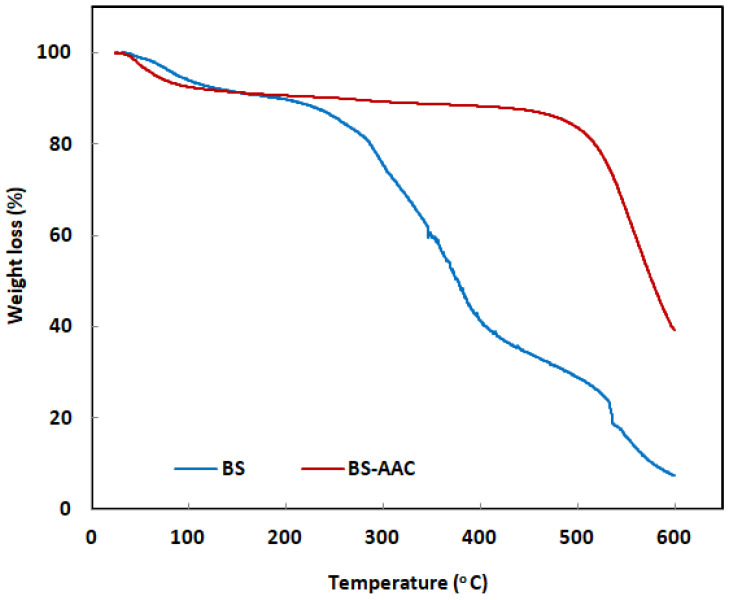
TGA curves of BS and BS-AAC.

The thermogravimetric analysis and resulting TGA were used to determine the thermal stability of BS and BS-AAC. As the temperature increased, the BS and BS-AAC TGA curves revealed a trend of weight loss (Figure 5). Due to the moisture adsorbed on the surface of biochar and functional groups, there was an 8.99% decrease in BS weight in the first stage, from 34.00 °C to 162.52 °C [24]. The 48.45% weight loss in the second stage from 162.52 °C to 396.88 °C could be explained by the thermal degradation of cellulose and hemicellulose content in BS [25]. The third stage demonstrates that pyrolysis processes continue to break down cellulose and hemicelluloses, as evidenced by a 30.37% weight loss from 396.88 °C to 566.80 °C [24]. The weight loss, on the other hand, was reduced by 14.87% in the first stage of a two-step degradation of BS-AAC due to the presence of functional groups and moisture adsorbed on the surface [24]. The weight loss in the second stage was 47.27% once the temperature reached 550 °C. The results show that the main components of the biomass-derived activated carbon are hemicelluloses, cellulose, lignin, and polycyclic aromatic compounds. If the temperature is between 220 °C and 315 °C, hemicelluloses might break down. The decomposition of cellulose and lignin occurs between 315 and 400 °C [24]. Additional temperature rises will result in the full destruction of the polycyclic aromatic structures [25]. This conclusion was also supported by our FTIR. With a 62.14% weight reduction in BS-AAC, the FTIR data demonstrated that the structure of the baobab seeds changed during pyrolysis, as evidenced by the disappearance of several functional groups.

### 2.2. Oxidation of MG over BS-AAC and BS-BAC

**Figure 6:**
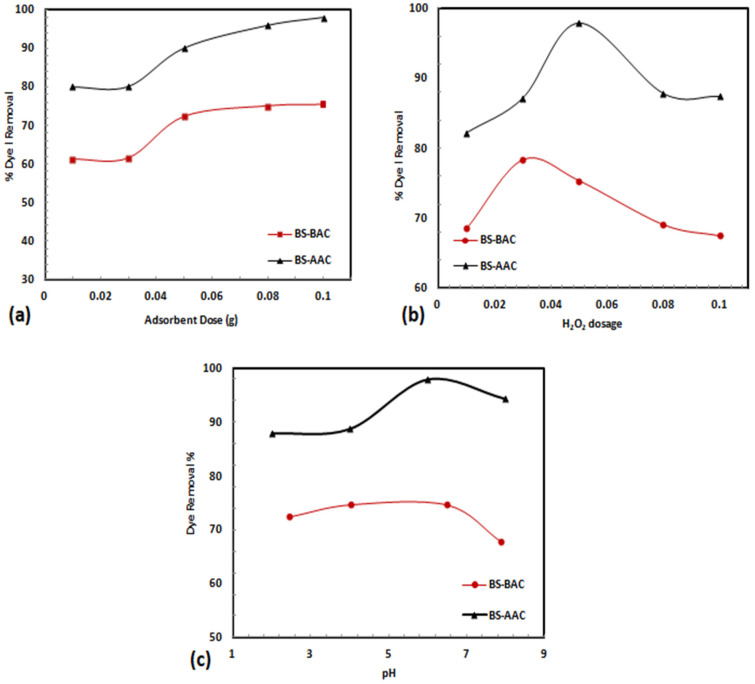
(**a**) Activated carbon dose effect (pH = 6.5, C_0_ = 10 mg/L, 0.05 M H_2_O_2_), (**b**) H_2_O_2_ dose effect (activated carbon dosage = 0.1 g, pH = 6.5, and C_0_ = 10 mg/L), and (**c**) pH effect (activated carbon dosage = 0.1 g, 0.05 M H_2_O_2_ (BS-AAC) and 0.03 M H_2_O_2_ (BS-BAC), and C_0_ = 10 mg/L).

To evaluate the oxidative degradation capacity of BS-AAC and BS-BAC, various conditions were examined such as activated carbon dosage, pH, and H_2_O_2_ concentration. The results presented in Figure 6 clearly demonstrate that the MG dye was effectively oxidized by hydroxyl radicals over both BS-AAC and BS-BAC. According to Figure 6a, increasing the activated carbon dosage from 0.03 to 0.05 g produced an increase in degradability in the MG over BS-AAC and BS-BAC by 72% and 90%, respectively. As the activated carbon dosage was increased from 0.05 g to 0.1 g, the oxidative degradation of MG was improved to 75% and 97.9%, respectively, for BS-AAC and BS-BAC. An increase in the number of active sites, which provided more active sides, could explain the increase in initial degradation with increasing activated carbon dosage [26,27].

Figure 6b illustrates the degradable ability of BS-AAC and BS-BAC for MG dye as a function of H_2_O_2_ dosage. According to studies, the degradation of MG dye increases with H_2_O_2_ dosage, reaching 97.9% (at 0.05 M H_2_O_2_) and 78% (at 0.03 M H_2_O_2_) for BS-AAC and BS-BAC, respectively. In both activated carbons, MG degradation was evidently steeply reduced above optimal levels of H_2_O_2_. Dye degradation rates increase with increasing concentrations of H_2_O_2_, which suggests that the radical •OH is primarily responsible for the dye degradation rate. As the H_2_O_2_ concentration increases, more radicals are created, which dimerize to propagate electron-hole recombination [26]. Studies have shown that the presence of excess H_2_O_2_ might result in the production of the unwanted hydroperoxyl radical HOO•, which can initiate a parallel process that may be self-scavenging due to its lower oxidation potential (1.780 eV) than the desired hydroperoxyl radical [26,28]. The BS-AAC and BS-BAC lose their oxidative capacity as a result of this phenomenon.

In the study of MG dye degradation over BS-AAC and BS-BAC, the impact of the solution pH was measured between 2 and 8 (Figure 6c), demonstrating that degradation increased significantly from 2 to 6, and BS-AAC and BS-BAC were ineffective in adsorbing malachite green at low pH values (2 and 4). The adsorption of positively charged dye cations was not preferred when the number of negatively charged adsorbent sites decreased, and the number of positively charged sites increased as the test solution’s initial pH decreased. This is because of electrostatic repulsion [29]. Reduced malachite green adsorption at an acidic pH is also caused by the presence of additional H^+^ ions that compete with dye cations for adsorption. However, the degradation then gradually declined above pH 6, sustaining an overall degradation of 68% and 94% for the MG dye over BS-BAC and BS-AAC, respectively (Figure 6c). Similar findings were reported by Navarro et al. [30], Rauf et al. [31], and Yong et al. [32], who found that MG degraded more quickly at pH 5 within the tested range (pH 5–8). This pattern may be connected to how pH affects the production of hydroxyl radicals. Because high pH causes H_2_O_2_ to deprotonate and form HO_2_^−^, which then interacts with H_2_O_2_ to produce dioxygen and water, low pH promotes MG decolorization because it limits the availability of hydroxyl radicals to attack the dye molecule [30].

### 2.3. Kinetic Study

**Figure 7:**
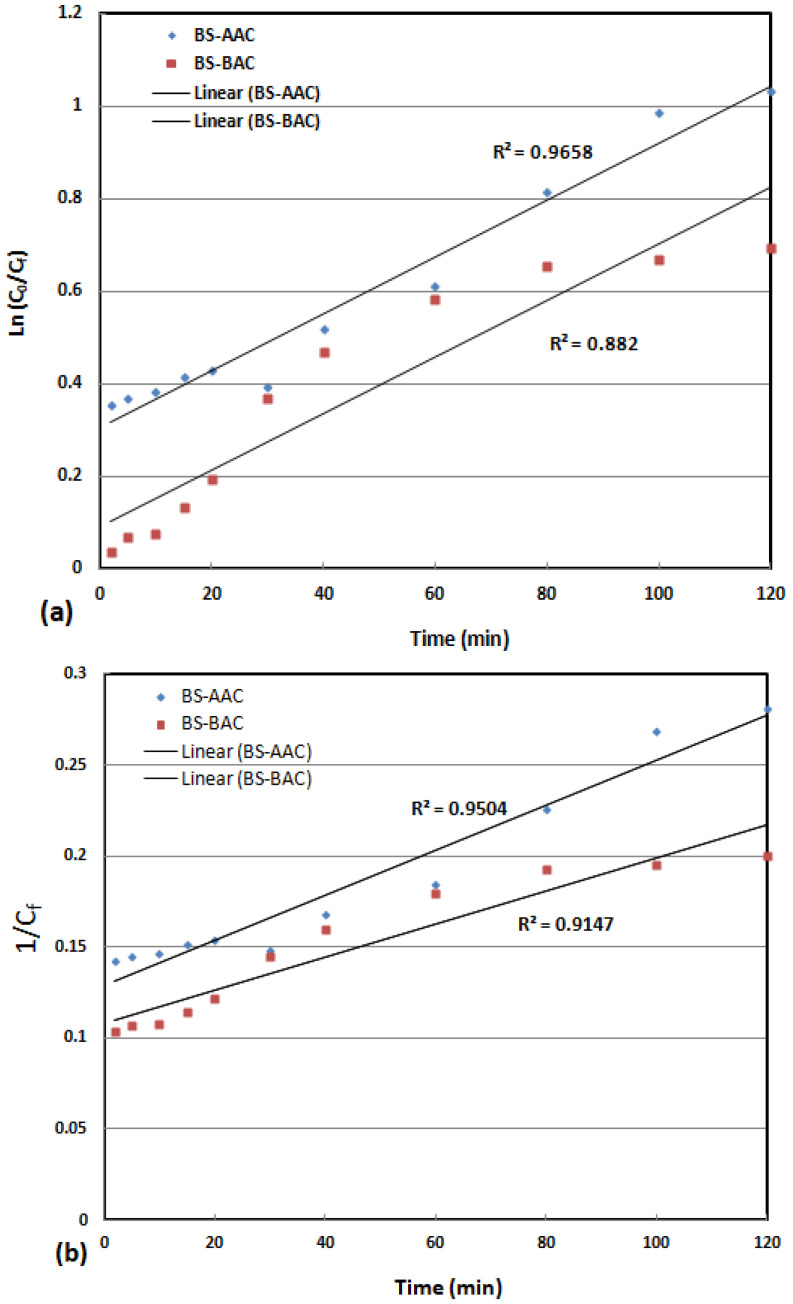
(**a**) A plot of ln(*C*_0_/*C_f_*) over time t (min) (*C*_0_ = 10 mg/L, activated carbon dosage = 0.1 g, and pH = 6.5), and (**b**) A plot of 1/*C_f_* over time t (min) (*C*_0_ = 10 mg/L, activated carbon dosage = 0.1 g, and pH = 6.5).

The color removal kinetics for MG dye were examined using pseudo-first-order (Equation (1)) and pseudo-second-order (Equation (2)) models [33]. The linearized version of these (Equations (3) and (4)) for the synthesis of activated carbons is displayed in Figure 7. Based on the models, rate constants *k*_1_ and *k*_2_ were calculated and are summarized in Table 3, as well as determination coefficient *R*^2^.

(1) $$ln\frac{C_{0}}{C_{f}}=k_{1}t$$

(2) $$\frac{1}{C_{f}}=k_{2}t+\frac{1}{C_{0}}$$

where *t* represents the time (min). The constants for the pseudo-first- and pseudo-second-order models are denoted by *k*_1_ and *k*_2_, respectively. For BS-AAC and BS-BAC, the first-order *R*^2^ is 0.966 and 0.882, respectively (Figure 7a). For the second order, the *R*^2^ for BS-AAC and BS-BAC is 0.950 and 0.915, respectively (Figure 7b), indicating that, for BS-AAC, the oxidation of MG dye is best described by the first-order kinetic model, while for BS-BAC, the second-order kinetic model is the most appropriate.

### 2.4. A Comparison of the Removal of MG Using Different Material

Because prior experiments employed different experimental conditions, it is challenging to directly compare the oxidation characteristics examined in this study with those previously published. However, as shown in Table 4, the activated carbon used in this study removes MG more efficiently than other materials [34,35,36,37,38].

### 2.5. Degradation Mechanism of MG Dye

In this work, a mechanism for decomposing H_2_O_2_ molecules and producing reactive species is proposed in the BS-AAC/H_2_O_2_ and BS-BAC/H_2_O_2_ systems. H_2_O_2_ molecules are adsorbed to active sites of activated carbon, which conducts heterogeneous reactions in the solid phase, leading to the generation of •OH (ads), as shown in Equation (3). The activated carbon surface adsorption of dye on its surface (Equation (4)) and H_2_O_2_ molecules facilitate the reaction between oxidant and active sites to generate free radicals in the solid state [40,41]. Finally, MG dye molecules are immediately attacked by OH• (ads) radicals, which causes degradation (Equation (5)).

AC + H_2_O_2_ → AC (OH•)_ads_ (H_2_O_2_ adsorption on the AC surface) (3)

Dye + AC → AC (dye)_ads_ (The dye’s adsorption on the AC surface) (4)

OH• + MG dye → degraded products(5)

### 2.6. Regeneration Studies

**Figure 8:**
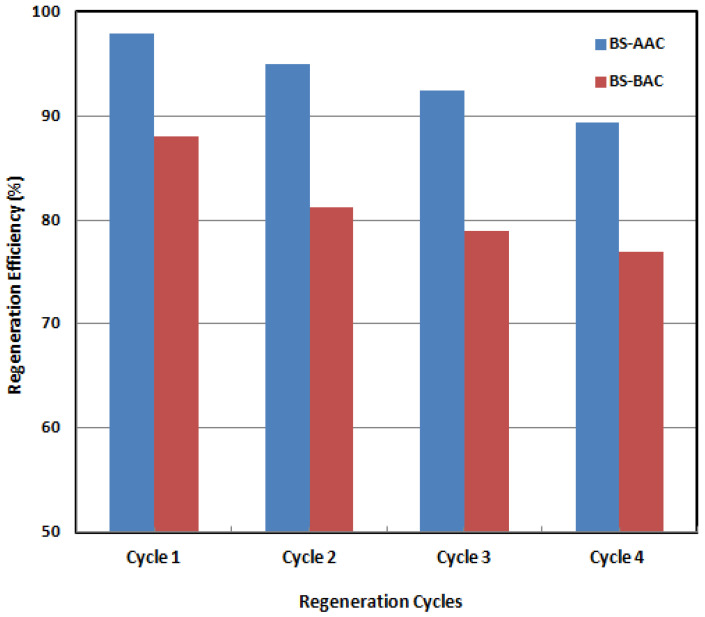
Regeneration studies of BS-AAC and BS-BAC for multiple degradation of MG.

Chemical regeneration eliminates pollutants from the activated carbon’s surface without causing carbon attrition by using certain chemicals [42,43]. The potential of this method is dependent on how soluble and reactive the pollutants are with the chemical reagents, which are used to dissolve pollutants using acid and base reagents [43]. After pollutants have been eliminated from the AC’s surface, the reagents must be eliminated in order to recover the regenerated AC [44,45]. To test activated carbons’ reusability, ACs loaded with dye were initially desorbed using 0.1 M NaOH. After that, distilled water and 0.1 M HCl were used to wash the air conditioners until the pH was about 7.0. AC regeneration and reuse studies were performed for the fourth cycles (Figure 8). Malachite green elimination was found to be above 89% for AC-AAC and 77% for AC-BAC, even after four regeneration cycles. The degradation efficiency of malachite green (MG) after four cycles is likely due to factors related to adsorption and oxidation processes. Adsorption-related issues include the loss of active sites, changes in porosity, and incomplete regeneration. Oxidation-related issues involve deactivation, oxidant depletion, and byproduct formation [43]. To improve the reusability of activated carbon, careful optimization of the regeneration process and an understanding of degradation mechanisms are crucial. Characterizing the activated carbon before and after each cycle can help identify the dominant factors contributing to the loss of efficiency.

## 3. Material and Methods

### 3.1. Material

**Figure 9:**
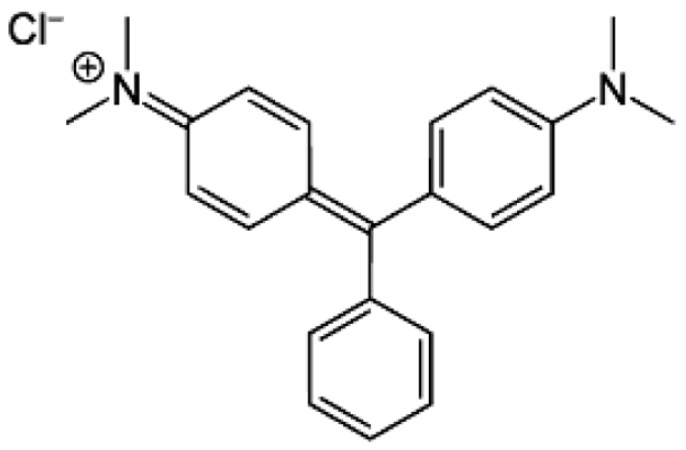
MG dye’s chemical structure.

The baobab seeds were collected from Khartoum market (Khartoum, Sudan). Sodium hydroxide (NaOH), sulfuric acid (H_2_SO_4_), and hydrochloric acid (HCl) were used for the production of AC. The solution pH was controlled using 0.1 M HCl and 0.1 M NaOH. Malachite green (98% MG) was used to evaluate the oxidation process. All the chemicals were purchased from Merck (Darmstadt, Germany). Figure 9 illustrates the MG dye’s chemical structure.

### 3.2. Synthesis of Activated Carbon

#### 3.2.1. Synthesis of AC Using Acid Activating Agent

**Figure 10:**
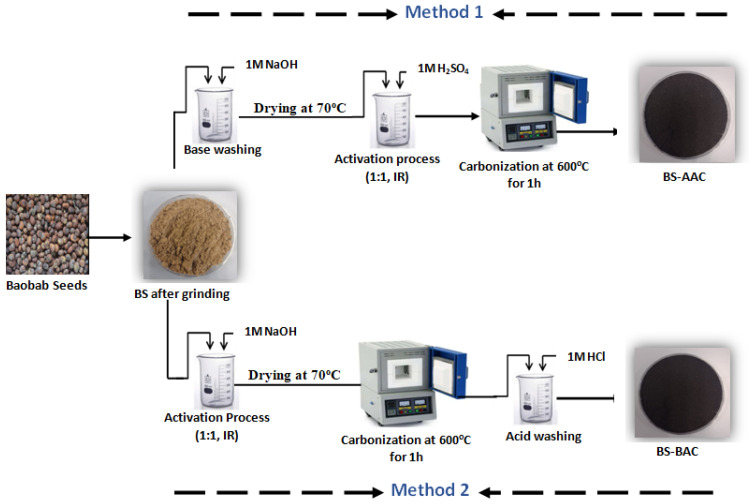
Synthesis of BS-AAC and BS-BAC.

For acid activation, the ground baobab seeds (BSs) were refluxed with 1 M NaOH solution for 1 h to reduce ash content in the sample. After the basic solution was drained, the baobab seeds were washed with distilled water and dried at 70 °C for 24 h. Subsequently, the baobab seeds were impregnated with 1 M H_2_SO_4_ at impregnation ratio (1/1, mass ratio of activating agent to dried baobab seeds). Then, the mixture was heated in a furnace with the absence of oxygen at 600 °C for 1 h. The selected temperature 600 °C was part of the input series applied to assess the best condition for the production of activated carbon from biomass per the literature [46,47]. The developed AC was named BS-AAC (Figure 10).

#### 3.2.2. Synthesis of AC Using Base Activating Agent

For base activation, the grinded baobab seeds were impregnated with 1 M NaOH at impregnation ratio (1/1, mass ratio of activating agent to dried baobab seeds). Then, the mixture was heated in a furnace with the absence of oxygen at 600 °C for 1 h. Then, the AC was washed with 1 M HCl at room temperature for one hour to remove the impurities. The washed samples were dried at 70 °C for 24 h. The developed AC was named BS-BAC (Figure 10).

### 3.3. Instrument Analysis

For the characterization of the samples, many techniques were utilized such as scanning electron microscopy (SEM, model FEI, QUANTA FEG, 250, FEI Company, Eindhoven, The Netherlands) to study the morphology of the BS-AAC and BS-BAC surface, and Brunauer–Emmet–Teller (BET) (ASAP 2020, Micromeritics, Norcross, GA, USA) was applied to investigate the surface and textural characteristics of the activated carbon produced. In addition, Fourier transform infrared (Cary 630 FTIR Spectrophotometer model, Agilent Technologies, Santa Clara, CA, USA) was used to specify the effective functional groups available on the surface of the BS-AAC and BS-BAC. Thermogravimetric–differential thermal analysis (TG–DTA, Perkin Elmer STA 6000, PerkinElmer, Waltham, MA, USA) was performed from 30 °C to 800 °C with heating rates of 10 °C per minute using N_2_ gas at 20 mL/min in order to determine sample weight loss and thermal stability. The yield of the ACs was determined using Equation (6).

(6) $$Activated\;carbon\;yield\left(\%\right)=\frac{mass\;of\;AC}{mass\;of\;dried\;sample}\times 100$$

### 3.4. Dye Oxidation

The oxidation process was evaluated by mixing 0.1 g of AC with 20 mL of a dye solution (10 mg/L) and H_2_O_2_ (0.05 M) at normal pH for 24 h at 120 rpm and 25 °C. The suspensions were separated using filter paper. In order to determine the dye concentrations in the filtrate, UV-Vis spectrophotometers (model UV-1700 Shimadzu, Shimadzu, Kyoto, Japan) were used. Based on the maximum absorbance of the dye solution at 617 nm, the dye concentration was calculated. In addition, the effect of various pHs (2–8), activated carbon dose (0.01–0.1 g), and H_2_O_2_ dose (0.01–0.1 M) was studied. The kinetic study was conducted at normal pH, 25 °C, and 120 rpm with an initial dye concentration of 10 mg/L, 0.1 g activated carbon dose, and a time interval of 2, 5, 10, 15, 20, 25, 30, 40, 50, 60, 80, 100, and 120 min. The dye removal efficiency was determined using Equation (7):

(7) $$Removal\;effeciency\left(\%\right)=\frac{\left(C_{0}-C_{f}\right)}{C_{0}}$$

where *C*_0_ represents the initial concentration of MG (mg/L), and *C_f_* represents the final concentration of MG (mg/L).

## 4. Conclusions

Baobab seeds were used to synthesize two activated carbons, BS-AAC and BS-BAC. The three materials were characterized in order to determine their morphological characteristics, functional groups, surface areas, and pore sizes. SEM analysis showed that activated carbon prepared from baobab seeds had a suitable morphology for dye oxidation. Equivalent FTIR spectra are available for BS, BS-ACC, and BS-BAC, showing different types of surface functional groups. BET analysis revealed that BS-AAC had the largest surface area (174.4 m^2^/g) with mesopores centered at 4 nm and 2.7 nm. Following physico-chemical analysis of both BS-AAC and BS-BAC, MG dye degradation was investigated at various activated carbon dosages, pH levels, and H_2_O_2_ concentrations, which are highly dependent on degradation efficiency. The degradable ability of BS-AAC and BS-BAC for MG dye as a function of H_2_O_2_ dosage was evidently steeply reduced above optimal levels of H_2_O_2_. BS-AAC and BS-BAC reached up to 97.9% and 78% MG removal, respectively, at the optimum level of H_2_O_2_. The oxidation of MG increased up to pH 6 and gradually declined above pH 6. For BS-AAC, a first-order kinetic model was fitted to the MG removal kinetic data, whereas for BS-BAC, a second-order kinetic model is appropriate. According to the results of this study, BS-AAC was found to be a more effective material than previously reported activated carbons for oxidizing MG dye from water solutions.

## Figures and Tables

**Table 1:** Elemental composition of BS, BS-AAC, and BS-BAC by EDS analysis.

Sample	Element				
	C%	O%	Si%	Na%	Cl%
**BS**	47.73	44.61	7.62	-	-
**BS-AAC**	86.44	9.93	2.55	0.88	-
**BS-BAC**	79.70	15.37	2.93	0.95	0.11

**Table 2:** Pore characteristics of BS-AAC and BS-BAC.

Adsorbent	BET Surface Area (m^2^/g)	Total Pore Volume ^a^ (cm^3^/g)	Average Pore Diameter ^b^ (nm)
**BS-AAC**	174.44	0.0630	6.24
**BS-BAC**	11.2	0.0107	62.30

^a^ Single-point adsorption total pore volume; ^b^ Adsorption average pore width (4V/A by BET).

**Table 3:** The correlation coefficient and constant parameters of kinetic models.

Model	Parameters	Sample	
		BS-AAC	BS-BAC
**Pseudo-first-order**	*k*_1_ (min^−1^)	0.0062	0.0061
	$R^{2}$	0.9658	0.8820
**Pseudo-second-order**	*k*_2_ (L/mg·min)	0.0012	0.0009
	$R^{2}$	0.9504	0.9147

**Table 4:** Comparison of the removal of MG using different material reported in the literature.

Material	Degradation	References
Bi_2_WO_6_ nanosheet	89%	[34]
BaO NPs	86.6%	[35]
Streptomyces chrestomyceticus S20	69.6%	[36]
Chitosan-based composites	54% and 87%	[37]
Pine needle biochar	85%	[38]
NiO/Al_2_O_3_	82%	[39]
BS-AAC BS-BBC	97.9% 78%	This work

## Data Availability

Data will be made available on request.
